# Cardiometabolic Heart Failure with Preserved Ejection Fraction (HFpEF): Epidemiology, Mechanisms, and the Role of Lifestyle Modification

**DOI:** 10.3390/jcdd13070291

**Published:** 2026-06-23

**Authors:** Daniel G. Yang, Shaleen Thakur, Harriet Akunor, Richard B. Stacey, Bharathi Upadhya

**Affiliations:** 1 Cardiovascular Medicine Section, Duke University School of Medicine, Durham, NC 27705, USA; daniel.yang@duke.edu (D.G.Y.);; 2 Department of Medicine, Icahn School of Medicine at Mount Sinai, New York, NY 10029, USA; 3 Department of Cardiovascular Medicine, Atrium Health Wake Forest, Winston-Salem, NC 27103, USA

**Keywords:** HFpEF, metabolic syndrome, obesity, exercise, diet

## Abstract

Heart failure (HF) with preserved ejection fraction (HFpEF) is increasingly prevalent and now recognized as a systemic syndrome with diverse clinical phenotypes and multiorgan involvement. The predominant clinical phenotype has evolved from patients with isolated hypertensive heart disease to individuals with cardiometabolic (CM) abnormalities [obesity, insulin resistance, increased waist circumference (a surrogate for visceral adiposity), dyslipidemia, type 2 diabetes, and hypertension] that result in metabolic alterations leading to CM-HFpEF. Indeed, CM-HFpEF and metabolic dysfunction-associated fatty liver disease are recognized as two sides of the same coin. Chronic systemic inflammation is a defining pathophysiologic feature of CM-HFpEF, with visceral adipose tissue serving as a central driver. In this regard, lifestyle changes, including diet and exercise, are crucial for managing HFpEF. Several recent studies have shown that exercise training (aerobic and resistance combined) with or without calorie restriction is an effective therapeutic management strategy for improving exercise capacity, physical function, and quality of life in patients with clinically stable HFpEF. Also, the pharmacologic interventions that have proven beneficial in HFpEF so far (sodium-glucose cotransporter 2 inhibitors and glucagon-like peptide-1 receptor agonists) are effective due to their metabolic protective effects. In this review, we outline the current available evidence on lifestyle interventions in HFpEF management and therapeutics, discussing their modalities and potential mechanisms.

## 1. Introduction

Heart failure (HF) with preserved ejection fraction (HFpEF) is the most common form of HF in the community, particularly among older persons and women [1,2]. Its prevalence continues to rise in parallel with an aging population and the global epidemics of obesity, type 2 diabetes (T2DM), and hypertension (HTN) [2]. HFpEF affects ~32 million people worldwide and represents over half of all HF cases. In high-income countries, prevalence is projected to exceed 8% in adults >65 years by 2030. In the USA, it accounts for ~50% of >6 million patients with HF and >1 million annual hospitalizations [3]. Unlike HF with reduced ejection fraction (HFrEF), for which multiple disease-modifying therapies have been established, HFpEF remains clinically challenging due to its heterogeneity, complex pathophysiology, and limited therapeutic options. Patients with HFpEF frequently present with multimorbidity, functional impairment, reduced quality of life (QoL), increased cardiovascular (CV) morbidity, and hospitalization worldwide [1,2]. During the last decade, our understanding of the pathophysiology (and our diagnostic approach) for HFpEF has markedly evolved, and HFpEF is recognized as a systemic syndrome characterized by multiorgan involvement, diverse clinical phenotypes, and intersecting CV and non-CV comorbidities [1,2].

Cardiometabolic (CM) disease is a cluster of interconnected conditions—central obesity, insulin resistance (IR), T2DM, HTN, dyslipidemia, and metabolic dysfunction-associated steatotic liver disease (MASLD)—that collectively drive systemic organ damage. These abnormalities often coexist as metabolic syndrome, characterized by chronic low-grade inflammation originating largely from visceral adiposity, which promotes coronary microvascular endothelial inflammation, oxidative stress, impaired nitric oxide (NO) bioavailability, and reduced cyclic guanosine monophosphate (cGMP)–protein kinase G (PKG) signaling, collectively driving myocardial hypertrophy, stiffness, and diastolic dysfunction (DD) [4,5,6,7,8]. Mounting evidence supports a dominant CM-HFpEF phenotype characterized by a cluster of the above-mentioned metabolic abnormalities, which contribute to a chronic pro-inflammatory state extending beyond the myocardium, affecting the vasculature, skeletal muscle (SM), and other organ systems [5,7,9]. Compared with other pathophysiological subtypes, CM-HFpEF is associated with a more severe symptom burden and reduced QoL [5,7,9]. Conventional HF therapies have shown limited efficacy in this phenotype, highlighting the need for a deeper understanding of their mechanisms and the development of targeted, phenotype-specific interventions. Important gaps persist in integrating epidemiology, systemic pathobiology, and lifestyle-centered therapeutics within a unified framework.

In this review, we examine CM-HFpEF as a systemic inflammatory-metabolic syndrome, outlining its epidemiological burden, clinical characteristics, and underlying molecular mechanisms. We then synthesize current evidence regarding lifestyle-based interventions—including dietary modification, weight management strategies, brief pharmacotherapy, and structured exercise training (ET)—highlighting their physiological rationale, clinical efficacy, and potential disease-modifying implications. By contextualizing lifestyle therapeutics within the broader mechanistic landscape of CM-HFpEF, we aim to clarify their role in contemporary management and identify key knowledge gaps that warrant future investigation.

## 2. What Has Driven CM-HFpEF to the Forefront of Public Health Concerns?

**Figure 1:**
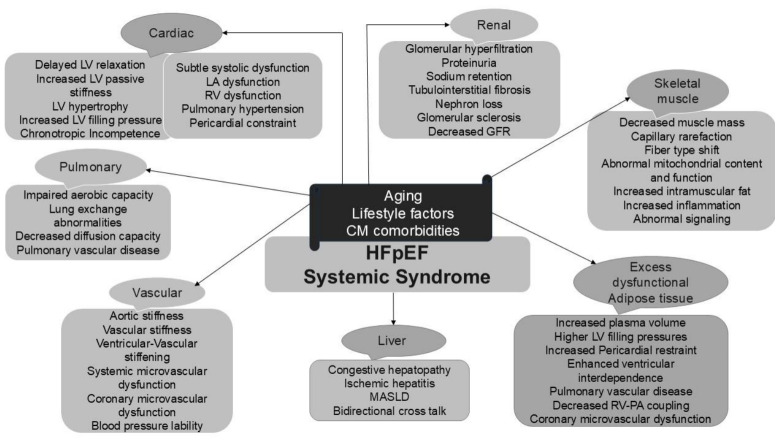
HFpEF is a systemic syndrome. Abbreviations: CM = cardiometabolic, GFR = glomerular filtration rate, HFpEF = heart failure with preserved ejection fraction, LA = left atrium, LV = left ventricle, MASLD = metabolic dysfunction-associated steatotic liver disease, PA = pulmonary artery, RV = right ventricle.

HFpEF was historically conceptualized primarily as a disease of LV DD; however, contemporary evidence has shifted this view toward a broader systemic framework [1,2,10]. Although DD is a prominent feature of HFpEF, the disease indisputably involves far more than just DD. Beyond myocardial involvement, HFpEF is associated with pathophysiologic changes across multiple organ systems, including pulmonary vascular abnormalities, renal dysfunction, SM impairment, and vascular stiffness. Thus, it must be considered a systemic syndrome (Figure 1) [1,2,5,6,7,8,10,11,12,13,14,15]. This change reflects a growing understanding that HFpEF arises from interactions among aging, lifestyle factors, genetic predisposition, and a wide spectrum of CM comorbidities [1,2,10]. Correspondingly, the predominant clinical phenotype has evolved from patients with isolated hypertensive heart disease to individuals with CM abnormalities [obesity, IR, increased waist circumference (a surrogate for visceral adiposity), dyslipidemia, T2DM, and HTN] that cause metabolic alterations leading to CM-HFpEF [1,2,3,5]. The epidemiology of this CM-HFpEF closely mirrors the worldwide increase in obesity and T2DM.

CM-HFpEF disproportionately affects older adults, with a higher prevalence among women, particularly those with central adiposity [16]. Visceral adiposity is the key feature defining metabolic syndrome and the central component of CM-HFpEF [7]. Over 80% of patients with HFpEF are either overweight or obese. In recent HFpEF trials, the average body mass index (BMI) of enrolled patients exceeded 36 kg/m^2^, and >90% had central obesity, as reflected by a waist-to-height ratio ≥ 0.5 [17,18]. Similarly, T2DM affects approximately 30–50%, with even higher rates observed in contemporary registries [19]. Higher BMI shows a clear dose−response relationship with increased risk of HFpEF, but not HFrEF, with the effect especially strong in women [20,21,22]. Multiple obesity metrics—including BMI, waist circumference, and visceral adiposity—independently predict incident HFpEF, as demonstrated in the Multi-Ethnic Study of Atherosclerosis cohort [23]. Similarly, racial and ethnic disparities have been observed, with increased burden among African American (AA) and Hispanic populations, likely reflecting both differential exposures to CM risk factors and social determinants of health (SDoH) [16]. Geographic variation aligns with regional differences in obesity and T2DM prevalence, underscoring the global nature of the syndrome [16].

Taken together, the combination of population aging and rising global prevalence of obesity, HTN, T2DM, and sedentary lifestyle behaviors ensures that the overall burden of CM-HFpEF will continue to grow and emerge as an expanding public health challenge [1,2,19,24]. CM-HFpEF is the most prevalent and deadly disease linked to metabolic alterations, having high morbidity with a 35% 2-year hospitalization rate and a 14% 2-year mortality rate, highlighting the need for population-level prevention strategies targeting modifiable metabolic risk factors [25]. CM-HFpEF and MASLD have been proposed to represent two manifestations of a shared CM continuum, reflecting both local and systemic expressions of common underlying mechanisms [26].

## 3. How Do Sex-Specific Biological and Hormonal Factors Influence the Development and Progression of CM-HFpEF?

Women are disproportionately affected by CM-HFpEF, with a nearly twofold higher lifetime risk of HFpEF than HFrEF, reflecting sex-specific differences in obesity, CM risk factors, and cardiac remodeling [27,28]. In CM-HFpEF, sex differences emerge both in how traditional CV risk factors affect cardiac function and in distinct pathophysiological mechanisms triggered by sex-specific events, such as menopause and adverse pregnancy outcomes [28]. Clustering of traditional CM risk factors is associated with a greater propensity for concentric left ventricular (LV) hypertrophy (LVH), DD, and incident HFpEF in women [28,29]. These differences are further amplified by sex-specific adipose tissue biology and inflammatory signaling, as well as reproductive and hormonal factors. Menopause-associated estrogen deficiency promotes visceral adiposity, metabolic dysfunction, vascular stiffening, and adverse cardiac remodeling [28]. Women with CM-HFpEF more commonly exhibit excess visceral and epicardial adiposity, systemic inflammation, coronary microvascular dysfunction, abnormal ventriculo-arterial coupling, and concentric cardiac remodeling, accompanied by smaller LV chamber size and more pronounced limitations in exercise capacity related to impaired cardiac reserve and peripheral oxygen extraction [28,29]. Pregnancy is a unique CM stressor that may unmask susceptibility to CM-HFpEF, particularly in women with obesity, HTN, T2DM, or adverse pregnancy outcomes [29,30]. DD and adverse LV remodeling can develop during pregnancy complicated by preeclampsia and persist postpartum, which is associated with approximately a 4-fold increased risk of future HF and a markedly higher risk of subsequent HTN [31]. Adverse pregnancy outcomes identify women at increased long-term CV risk [28]. This supports pregnancy as an early window to identify women at increased long-term risk of HFpEF and target preventive strategies [30,31]. Taken together, these mechanisms contribute to a distinct HFpEF phenotype in women and highlight the importance of sex-specific approaches to risk assessment, phenotyping, and therapeutic development.

## 4. How Do SDoH Contribute to Disparities in Incidence and Prognosis of CM-HFpEF?

SDoH may drive CM-HFpEF by shaping the development of CM risk factors. Limited education, food insecurity, low income, inadequate access to healthcare, and living environments that discourage physical activity can contribute to higher rates of obesity, HTN, and T2DM [32,33]. Furthermore, factors such as poverty, limited access to healthcare and healthy foods, unsafe living environments, social isolation, discrimination, and adverse childhood experiences contribute to HF development through chronic psychosocial stress, inflammation, neurohormonal activation, endothelial dysfunction, and adverse health behaviors [32,33]. In addition, SDoH have been increasingly recognized as critical determinants of racial disparities in HF health outcomes [34]. SDoH often lead to delay in seeking medical attention, reduced medication adherence, and worse clinical outcomes, making HF not only a biological disease but also one strongly molded by socioeconomic and structural factors that affect health across the lifespan [32,33]. The association between SDoH and incident HFpEF is biologically plausible, consistent across multiple studies, and likely mediated by both traditional CV risk factors and chronic stress and inflammatory pathways. Recently, in a cohort of patients with HFpEF at a large multihospital system, lack of commercial medical insurance, rurality, and AA race were associated with worse clinical outcomes [35]. Collectively, these findings support the incorporation of social and psychosocial determinants as important risk enhancers in CM-HFpEF prevention and management strategies [32,33]. ACC/AHA/HFSA guidelines and the 2024 expert consensus decision pathway for HF highlight the importance of SDoH in the management of HF [3,36].

## 5. What Are the Mechanistic Underpinnings of CM-HFpEF?

**Figure 2:**
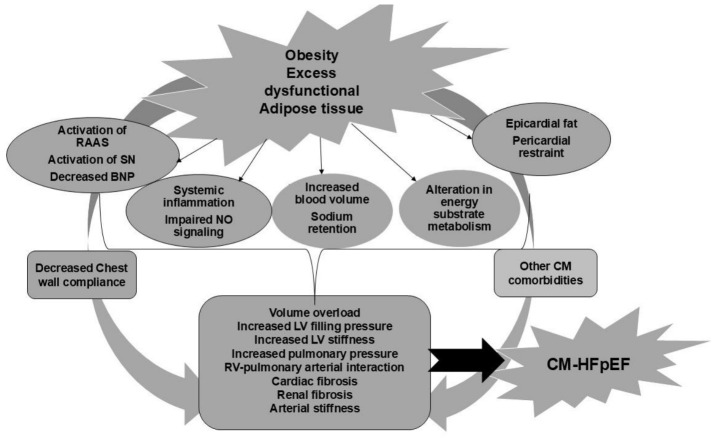
Visceral adiposity is essential in the pathogenesis of CM-HFpEF. Abbreviations: BNP = B-type natriuretic peptide, CM = cardiometabolic, HFpEF = heart failure with preserved ejection fraction, LV = left ventricle, NO = nitric oxide, RAAS = renin–angiotensin–aldosterone system, RV = right ventricle, SN = sympathetic nervous system.

Chronic systemic inflammation is a defining pathophysiologic feature of CM-HFpEF, with visceral adipose tissue serving as a central driver [4,6,7,37]. CM comorbidities, along with aging, promote chronic systemic inflammation that drives coronary microvascular endothelial inflammation, oxidative stress, impaired NO bioavailability, and reduced cGMP-PKG signaling, collectively driving myocardial hypertrophy, stiffness, and DD [4,8,15,38]. Neurohormonal perturbations, including elevated leptin and aldosterone levels and diminished NP signaling, further drive prohypertrophic and profibrotic remodeling, as reflected clinically by exercise-induced elevations in cardiac troponin [12]. In addition, excess adipose tissue increases mechanical strain on the heart and exacerbates comorbidities like IR and HTN. Adipose tissue is also highly metabolically active and exerts detrimental effects on CM health by directly influencing cardiac metabolism and immune activation, and by dictating inflammatory responses in HFpEF (Figure 2) [5,6,7,9,13]. Lipid dysregulation associated with CM-HFpEF impairs cardiomyocyte metabolism by shifting substrate utilization from fatty acids to carbohydrates under stress to maintain cardiac efficiency [39]. This results in lipid accumulation, lipotoxicity, and mitochondrial dysfunction, which exacerbate myocardial energetics. Beyond myocardial involvement, CM-HFpEF is associated with pathophysiologic changes across multiple organ systems (kidney, liver, SM), driven by chronic inflammation, metabolic dysregulation, and endothelial dysfunction [8,12]. Collectively, these mechanisms position chronic systemic inflammation and multiorgan dysfunction as central drivers of CM-HFpEF pathophysiology, linking comorbidities to myocardial remodeling and dysfunction. Despite strong mechanistic associations, its role as a direct therapeutic target remains incompletely defined in clinical practice. Better characterization of inflammatory pathways may help resolve the “unsolved puzzle” of CM-HFpEF heterogeneity and progression [40].

Although generalized and visceral adiposity are essential in the pathogenesis of obesity-related HFpEF via multiple mechanisms (Figure 2), there is increasing recognition of the potential role of epicardial adipose tissue (EAT) in disease pathogenesis [7,12,41]. EAT is a metabolically active tissue between the visceral pericardium and the underlying myocardium [11]. Expansion and increased secretion of cytokines from the EAT have been proposed as mechanisms contributing to meta-inflammation in HFpEF [12]. Echocardiographic EAT thickness ≥ 8 mm over the right ventricular (RV) free wall at end-diastole is widely used as a high-risk threshold, derived from observational CM and echocardiographic studies [42]. However, guidelines do not formally endorse it. Although echocardiography is the most practical modality for routine assessment, CMR and CT provide more accurate volumetric quantification, with EAT volume showing a stronger association with CM risk and clinical outcomes than EAT thickness [43,44,45]. In HFpEF, greater EAT burden is consistently associated with adverse outcomes, including HF hospitalization, CV death, and all-cause mortality, independent of BMI and established risk markers [43,44,45]. Increased EAT is associated with increased markers of systemic inflammation and myocardial injury, greater elevation in cardiac filling pressures, more severe pulmonary hypertension (PH), and greater pericardial restraint, culminating in poorer exercise capacity, worse left atrioventricular and RV right ventriculo-arterial coupling, and increased hazards for adverse events in HFpEF [41,44].

## 6. How Is CM-HFpEF Different from Non-CM-HFpEF?

**Figure 3:**
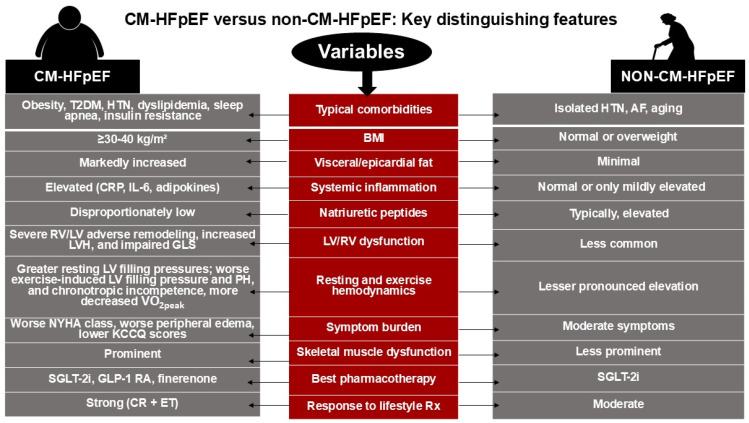
CM-HFpEF versus non-CM-HFpEF: key distinguishing features. Abbreviations: CM = cardiometabolic, HFpEF = heart failure with preserved ejection fraction, T2DM = diabetes, HTN = hypertension, AF = atrial fibrillation; BMI = body mass index, CRP = C-reactive protein; IL-6 = interleukin 6, LV = left ventricle, RV = right ventricle, LVH = LV hypertrophy, GLS = global longitudinal strain, PH = pulmonary hypertension, VO_2peak_ = peak oxygen consumption, NYHA = New York Heart Association, KCCQ = Kansas City Cardiomyopathy Questionnaire, SGLT-2i = sodium-glucose cotransporter-2 inhibitor, GLP-1 RA = glucagon-like peptide-1 receptor agonist, CR = caloric restriction, ET = exercise training, Rx = intervention.

Traditional HFpEF was historically conceptualized as “hypertensive heart disease”—a condition driven primarily by chronic pressure overload, concentric LVH, and isolated DD in older patients with systolic HTN. While HTN remains prevalent in CM-HFpEF and contributes to its pathophysiology, CM-HFpEF is a phenotypically and mechanistically distinct entity [7,9,46,47]. In contrast to hypertensive HFpEF, CM-HFpEF is defined by the additional presence of visceral obesity, IR, and systemic metabolic inflammation—drivers that operate largely independently of blood pressure [7,9,46,47]. Figure 3 shows the key distinguishing features between CM-HFpEF and non-CM-HFpEF endotypes across clinical, hemodynamic, structural, and therapeutic domains. Structurally, CM-HFpEF is characterized by greater RV dysfunction, increased epicardial adipose tissue, higher blood volume, and biventricular volume overload, with adverse biventricular remodeling [7,9,46,47]. This can stretch the pericardial sac, leading to a steeper pressure–volume relationship, which enhances diastolic ventricular interaction and pericardial restraint [7,9]. In contrast, LV diastolic parameters and LVEF showed no difference between CM and non-CM-HFpEF endotypes. Importantly, the inflammatory-metabolic pathway in CM-HFpEF—driven by adipokine excess, endothelial microvascular inflammation, and lipotoxicity—is not present in the classic hypertensive phenotype, which tends to respond better to conventional antihypertensive and neurohormonal therapies [7,9,46,47]. This mechanistic distinction helps explain why CM-HFpEF responds differently to treatment and why targeted metabolic and anti-inflammatory strategies [sodium-glucose cotransporter 2 inhibitors (SGLT2i) and glucagon-like peptide-1 receptor agonists (GLP-IRA)] and lifestyle modification are particularly effective in this subtype. In addition to all these factors, elevated BMI can also challenge HF diagnosis by lowering the sensitivity of clinical history and physical examination. It is also associated with reduced NP levels, further complicating the detection of HF.

## 7. Can Lifestyle Interventions Modify the Course of CM-HFpEF?

Dietary modification, weight management strategies, and structured ET have demonstrated improvements in functional capacity, symptoms, and QoL in multiple clinical studies in HFpEF [1,15,32,48,49]. As such, sustained adherence to dietary and lifestyle measures should be regarded as a central pillar of disease-modifying therapy in this population. The question is no longer whether diet matters in HFpEF, but how to deploy it with precision.

Thinking beyond sodium restriction requires a paradigm shift: While relevant, focusing solely on sodium restriction is reductionist in HFpEF; congestion is only one facet of a broader systemic disorder. Even regarding sodium restriction, however, concerns exist about neurohormonal activation and decreased renal perfusion if dietary sodium restriction is too strict [50]. To date, the only relevant trial conducted solely in patients with HFpEF randomized 53 hospitalized patients (age 72 ± 12 years, 68% women) to aggressive sodium (800 mg/d) and fluid (800 mL/day) restriction, with no difference in weight loss (WL) or congestion score noted over 7 days, with an increase in thirst and reduction in calorie intake in the intervention group [51]. Current guidelines state that the restriction is “reasonable” for symptomatic HF, reflecting limited supporting evidence [36].

## 8. Can Dietary Modification Improve Outcomes in CM-HFpEF?

Among dietary regimens, the Dietary Approaches to Stop Hypertension (DASH) diet [fruits, vegetables, whole grains, nuts and legumes, low-fat dairy, and lean protein] has shown a trend towards benefit in HFpEF. Hummel et al. studied 13 patients with stable HFpEF (age, 72 ± 10 years; predominantly women; BMI, 36 ± 8 kg/m^2^) with HTN on a sodium-restricted DASH diet (target sodium, 1150 mg/2100 kcal) for 21 days, lowering clinic and ambulatory blood pressure. It also improved DD, arterial stiffness, ventricular–arterial coupling, and 6 min walk distance (6MWD) [52,53]. In the GOURMET-HF (Geriatric Out-of-Hospital Randomized Meal Trial in HF) trial, 4 weeks of home-delivered sodium-restricted DASH meals in 66 patients (age, 71 ± 8 years; 30% women; BMI, 33 ± 8 kg/m^2^; EF, 39 ± 18%, 1500 mg sodium/day) versus usual care showed a favorable trend in rehospitalization in 30 days [54]. In participants with HFpEF (LVEF ≥ 50%), the Kansas City Cardiomyopathy Questionnaire summary score (physical limitations and symptoms domains) improved between baseline and 4 weeks in the patients receiving meals, but not in the usual care group [54].

To date, no randomized controlled trials have been designed to investigate the impact of the Mediterranean diet (MedDiet), which has similarities to the DASH diet; it differs by emphasizing the liberal use of extra-virgin olive oil rich in unsaturated fatty acids (UFAs), and wine, typically red, is consumed in moderation with meals. Adherence to the MedDiet did not influence long-term mortality after an episode of acute HF in 991 patients (age, 80 ± 10 years; 58% women; BMI, 28 ± 5 kg/m^2^; EF, 51 ± 14%; 53% of whom were adherent to the MedDiet). However, it was associated with decreased rates of rehospitalization during the next year [55]. Plant-based diets may improve CM risk factors in HFpEF, but their direct impact on cardiac outcomes remains unclear [56,57]. Ketogenic diets may improve HFpEF features and exercise tolerance, but they involve potential cardiac risks, and definitive clinical evidence remains lacking [56,57]. Intermittent fasting (time-restricted eating) may modestly reduce energy intake without major dietary changes, but evidence in HFpEF is lacking [58]. Table 1 presents randomized controlled trials (RCTs) evaluating other oral macro- and micronutrient supplementation in patients with HFpEF. Table 2 shows the ongoing RCTs of dietary interventions in patients with HFpEF.

## 9. What Is the Role of Weight Loss (WL) and Calorie Restriction (CR) in CM-HFpEF?

**Figure 4:**
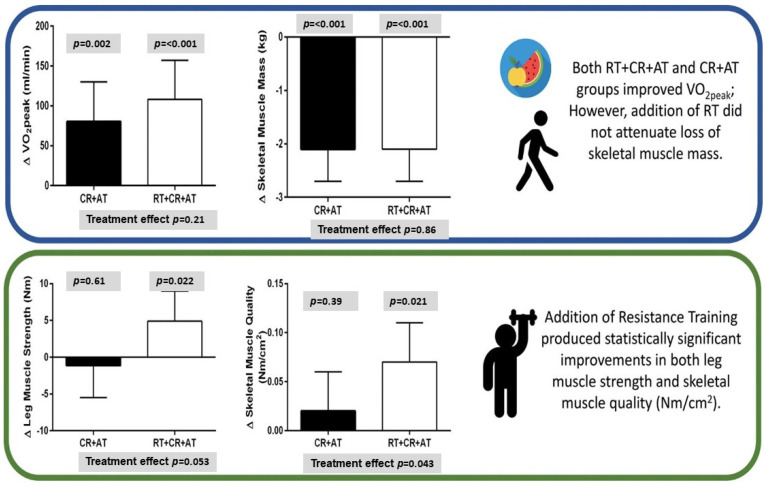
Effects of RT added to CR plus AT in CM-HFpEF. Effect sizes for the primary (VO_2peak_) and key secondary outcomes (strength and body/muscle composition from baseline to 20-week follow-up). Abbreviations: AT = aerobic exercise training, CM = cardiometabolic, CR = caloric restriction, HFpEF = heart failure with preserved ejection fraction, RT = resistance training, VO_2_ = oxygen consumption. Adapted from Ref. [49].

Weight management represents a central therapeutic target in CM-HFpEF, given the strong mechanistic link between obesity, visceral adiposity, and systemic inflammation. Among 100 older adults with obesity (age, 67 ± 5 years; 81% women; BMI, 39 ± 6 kg/m^2^) and HFpEF who were in a 2 × 2 factorial design to CR, supervised ET (SET), diet + SET, or attention control (AT), CR-induced WL improved exercise capacity to a degree comparable to SET, with combined intervention producing additive gains in functional capacity [48]. CR is also associated with greater improvements in QoL than SET alone [48]. CR was approximately 400 kcal/d for the diet group and approximately 350 kcal/d for the exercise + diet group (the difference between the groups allowed for the energy expenditure of the exercise intervention), but not less than 1000 kcal/d. The diet provided approximately 1.2 g of protein/kg ideal body weight, 25% to 30% of calories as fat, and the remainder as carbohydrate [48]. Increase in peak oxygen consumption (VO_2peak_) was also associated with fat mass and high-sensitivity C-reactive protein (CRP) [48]. Adding resistance training (RT) to CR + AT among 88 older adults with HFpEF (age, 68 ± 5 years; 85% women; BMI, 40 ± 6 kg/m^2^) increased leg strength and muscle quality without attenuating SM loss or further increasing VO_2peak_ or QoL (Figure 4) [49]. Despite an increase in relative SM percentage, absolute SM mass declined modestly (~2.1 kg) without between-group differences, indicating RT does not fully offset muscle loss during energy restriction [49]. In a diverse population of 41 patients with obesity (age, 67 ± 9 years; 66% women; mean BMI, 41 kg/m^2^) and HFpEF, energy restriction and unsupervised exercise improved 6MWD and QoL with improvements persisting for at least 3 months after intensive lifestyle interventions ended [70].

Given that >80% of patients with HFpEF are overweight or obese, these observations underscore intentional WL as a central component of therapeutic management in this population. Notably, however, weight regain frequently occurs after cessation of intensive lifestyle intervention or after discontinuation of the GLP-1RA. A recent long-term follow-up (28.0 ± 10.8 months) of the RCT of the CR and ET trial in obese patients with HFpEF demonstrated that a ∼5 kg weight regain after completion of the intervention, composed mostly of fat mass, was associated with a worsened lean-to-fat mass ratio compared with preintervention, and increased fat mass associated with an inverse trend with VO_2peak_ over time [71]. This suggests a need for long-term adherence strategies to prevent weight regain following CR in patients with CM-HFpEF.

## 10. What Is the Role of Bariatric Surgery (BSx) in Preventing or Improving HFpEF?

Retrospective cohort studies consistently show that surgical WL is associated with ~50% lower risk of incident HF across diverse comparator groups. However, incident HF was not phenotyped in these analyses. Thus, whether BSx surgery preferentially prevents HFpEF or HFrEF remains unclear [72,73]. In one retrospective BSx cohort of 213 patients (age, 54 ± 11 years; 67% women; BMI, 45 ± 10 kg/m^2^; LVEF > 50%), WL was associated with epicardial fat reduction, reduced ventricular interaction, LV reverse remodeling, and improved longitudinal biventricular mechanics (assessed by echocardiography). However, left atrial remodeling and dysfunction progressed, with higher atrial fibrillation prevalence and elevated filling pressures (E/e′). These findings suggest HFpEF risk is not fully mitigated by WL, underscoring the need for randomized trials assessing cardiac and clinical outcomes [74]. In a small cohort study (n = 12; all women; mean age, 47 years; BMI 44 ± 1 kg/m^2^), BSx-induced WL in women with obesity-related HFpEF improved symptoms, reversed adverse LV remodeling, enhanced diastolic function, and was associated with changes in the plasma lipidome [75]. The findings are limited by the very small sample size and the short follow-up duration. BSx was associated with lower all-cause mortality, fewer HF hospitalizations, and reduced atrial fibrillation in a real-world, observational cohort of 298,101 Medicare-enrolled patients with HF (55% women; age, 56 years; BMI, 52 kg/m^2^). Benefits were consistent across both HFpEF and HFrEF, suggesting WL may improve outcomes throughout the HF spectrum [76]. Despite its benefits, patients with lower socioeconomic status were less likely to undergo surgery, highlighting disparities in access and utilization.

There are currently no clinical trials of BSx in patients with established HF. Data on HFpEF are even more limited, partly because diagnostic criteria are less sensitive in patients with severely increased BMI.

## 11. How Does Physical Activity Influence Functional Capacity in CM-HFpEF?

Physical inactivity is associated with subclinical abnormalities in cardiac structure and function—including worsening DD and increased LV stiffness—that precede overt HFpEF [21]. Sedentary behavior promotes CM derangements—including insulin resistance, impaired energy utilization, endothelial dysfunction, and oxidative stress—that converge to increase myocardial stiffness, LVH, DD, and susceptibility to HFpEF [32]. ET is among the most consistently effective lifestyle interventions for patients with HFpEF, particularly for ameliorating exercise intolerance, a central feature of the syndrome [1,77]. Multiple trials demonstrate that ET improves exercise capacity, 6MWD, and QoL in HFpEF [1,77,78]. A recent review of 11 RCTs confirmed that SET [aerobic, high-intensity interval training (HIIT), resistance, and home-based programs] enhances functional capacity, with ~14% increases in VO_2peak_ versus slight declines in controls; by comparison, a 6–7% increase is clinically meaningful in HFrEF [78]. However, effects on QoL, diastolic function, and CV parameters remain inconsistent across studies [78]. Despite this heterogeneity, the available evidence supports SET as a central nonpharmacologic strategy in HFpEF, given the limited efficacy of current therapies [78]. A meta-analysis showed that HIIT was the best exercise modality for improving VO_2peak_ and QoL over about 16 weeks, followed by low-intensity training with a low-calorie diet in terms of effectiveness [17]. However, trials like optimizing ET in the prevention and treatment of diastolic HF reported no superiority of HIIT over moderate intensity continuous training (MICT) or guideline-based activity in HFpEF [79]. Table 3 shows all the ET clinical trials in HFpEF. In addition, SET is safe, and no major adverse events occurred in most clinical trials [1,77,78,80].

**Figure 5:**
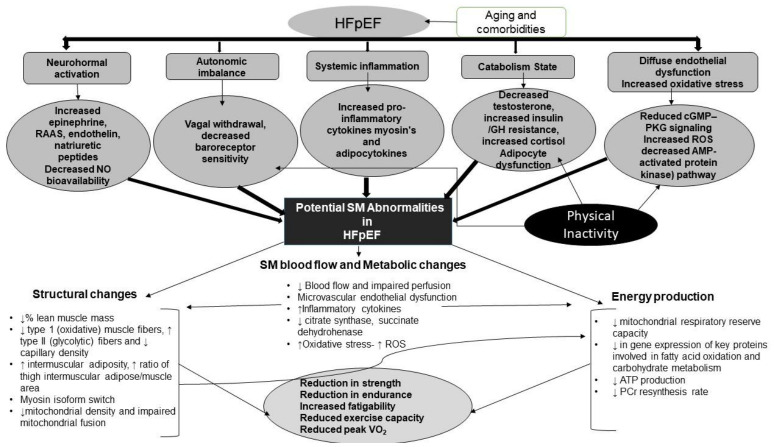
Potential causes for the SM abnormalities in CM-HFpEF. ↓ = decreased; ↑ = increased; Abbreviations: AMP = adenosine monophosphate, ATP = adenosine triphosphate, cGMP = cyclic guanosine monophosphate, CM = cardiometabolic, GH = growth hormone, HFpEF = heart failure with preserved ejection fraction, NO = nitric oxide, PCr = phosphocreatine, PKG = protein kinase G, RAAS = renin–angiotensin–aldosterone system, ROS = reactive oxygen species, SM = skeletal muscle, VO_2_ = oxygen consumption. Adapted from Ref. [105].

While the improvement in exercise capacity with ET is due in part to “noncardiac” peripheral adaptations (e.g., improved SM function) in both HFrEF and HFpEF, the relative contribution of peripheral adaptations to improvements in exercise intolerance appears to be much more prominent in patients with HFpEF [1,77,103,104]. ET enhances cardiac, pulmonary, vascular, and SM function, thereby improving oxygen delivery and utilization during physical activity [1,103,104]. Because exercise intolerance in HFpEF is largely driven by impaired oxygen transport and SM dysfunction, these physiological adaptations directly address key mechanisms underlying functional limitation [1,77,103,104]. Emerging evidence indicates that patients with CM-HFpEF exhibit a broad spectrum of SM abnormalities, which are strongly associated with impaired exercise capacity [1,77,104]. As illustrated in Figure 5, these alterations encompass structural, metabolic, and functional derangements within SM [1,77,104]. These abnormalities likely represent intrinsic components of the CM-HFpEF syndrome rather than secondary epiphenomena, arising from chronic systemic inflammation, neurohormonal activation, and impaired SM perfusion [1,77,104]. ET is also anti-inflammatory [1,77,104,105]. In a post hoc analysis of the ET in diastolic HF, ET was associated with increased endogenous ghrelin levels, a growth hormone-releasing peptide, which have mainly been attributed to the metabolic system and changes in body composition [105].

Despite multiple benefits of ET, the Centers for Medicare & Medicaid Services reimburse for exercise-based cardiac rehabilitation only for HFrEF after 4 weeks of post-hospitalization stabilization [106]. Patients with HFpEF were explicitly excluded from Medicare coverage. Recently, a progressive, multidomain physical rehabilitation intervention started as early as possible following HF hospitalization among older patients with high comorbidity burden (age, 73 ± 8 years; 52% women; BMI, 33 ± 8 kg/m^2^), severe physical dysfunction, and high rates of frailty showed it was feasible, safe, and resulted in improved physical function, frailty, QoL, and depression [95]. Also, those with HFpEF had significantly worse impairments at baseline and derived greater benefit from the intervention [107].

## 12. What Is the Best Exercise Prescription?

Best practices for ET “prescription” in CM-HFpEF remain an area of active research. Practical exercise prescriptions should consider baseline functional capacity and frailty to minimize injury risk. A supervised maximal exercise test with monitoring for ischemia should be performed before patients with HFpEF begin an ET program. For outpatients with stable, compensated HFpEF, MICT is the preferred first-line exercise strategy. It should be initiated under supervision, with gradual progression to activities such as walking, cycling, or elliptical training, for 3–4 sessions/week, starting with 10–30 min at 30–50% heart rate reserve (HRR), building to 45–60 min at 50–60% HRR (Borg rate of perceived exertion between 10 and 14 out of 20), with reassessment every ~3 weeks, and advancement as tolerated [80]. Frail or sedentary patients may benefit from shorter, more frequent exercise sessions initially [1,80,95]. Transition to home-based ET is a vital part of the long-term exercise prescription. Incorporation of RT may further enhance muscle strength and functional capacity. RT should range from 40% to 60% of maximal strength for one set, 2–3 days per week [77,80]. Combining ET with dietary interventions may also augment improvements in exercise performance [80].

## 13. What Is the Clinical Value of Cardiopulmonary Exercise Testing (CPET) in Guiding Therapeutic Interventions and Monitoring Treatment Response in CM-HFpEF?

In HFpEF, CPET characterizes functional limitation and guides individualized management. Integrating variables such as VO_2peak_, oxygen pulse, and the ventilatory equivalent for carbon dioxide (VE/VCO2) slope enables phenotyping of exercise intolerance and cardiopulmonary reserve, with VO_2peak_ reflecting aerobic capacity, oxygen pulse offering insight into stroke volume response and functional adaptation, and VE/VCO2 slope indicating ventilatory inefficiency that is often associated with more advanced disease or concomitant pulmonary vascular pathology. These CPET-derived profiles have both diagnostic and prognostic relevance, particularly in patients with unexplained exertional dyspnea and inconclusive resting evaluations, and can inform tailored interventions. For example, patients with elevated VE/VCO2 slope may benefit from structured, supervised aerobic exercise programs with gradual progression, often combined with RT and potentially MICT to improve functional capacity and ventilatory efficiency. Overall, CPET shifts HFpEF management toward personalized functional assessment and targeted rehabilitation strategies, though further evidence is needed to standardize its prognostic use [108].

## 14. How Do Emerging Pharmacological Therapies Improve CV Outcomes in Patients with CM- HFpEF?

SGLT2i have emerged as an important therapy for CM-HFpEF, providing substantial CV benefits beyond glycemic control. In patients with HFpEF, with or without T2DM, SGLT2i consistently reduce the risk of HF hospitalization and CV death, improve health status and QoL measures, increase exercise capacity, and reduce functional limitations [109,110,111,112,113]. Mechanistically, the benefits of SGLT-2i in CM-HFpEF likely reflect their metabolic protective properties: reduction of visceral and epicardial fat mass, improvement in myocardial energetics, mitigation of the pro-inflammatory adipokine milieu, decreased neurohormonal activation, improved coronary microvascular dysfunction, improved endothelial function, improved mitochondrial respiration, mitigation of myocardial fibrosis—all central drivers of CM-HFpEF pathophysiology—in addition to reduced cardiac preload and afterload through osmotic diuresis, lowered blood pressure, decreased arterial stiffness and preservation of renal function [109,110,111,112,113,114]. Collectively, these pleiotropic effects support the central role of SGLT2i in contemporary phenotype-directed management of CM-HFpEF.

**Figure 6:**
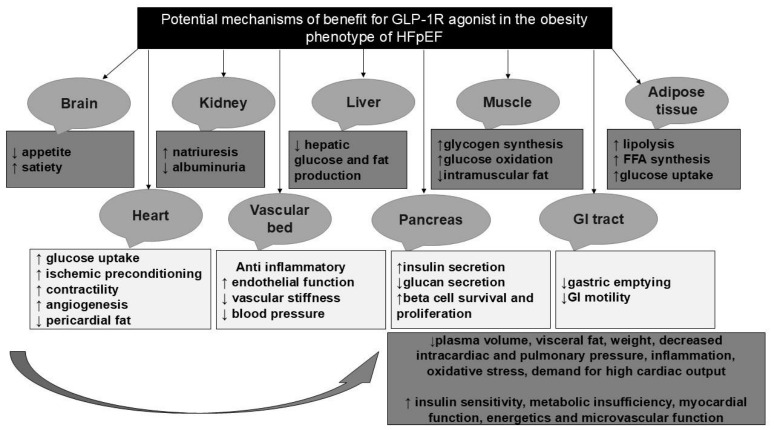
Potential mechanisms of benefit for GLP-1R agonists in the CM-HFpEF. ↓ = decreased; ↑ = increased; Abbreviations: CM = cardiometabolic, FFA = free fatty acid, GI = gastrointestinal, GLP-1R = glucagon-like peptide-1 receptor, HFpEF = heart failure with preserved ejection fraction.

GLP-1RAs have emerged as a promising therapeutic strategy for CM-HFpEF. Figure 6 shows the potential beneficial effect of GLP-1RA in CM-HFpEF. Among patients with HFpEF and obesity (BMI ≥ 35 kg/m^2^), treatment with semaglutide (GLP-1RA) led to greater reductions in symptoms, greater improvements in exercise function, and greater weight loss [115]. Also, the results of STEP-HFpEF (Semaglutide Treatment Effect in People with Obesity and HFpEF and Diabetes Mellitus) trials with semaglutide, which resulted in increased 6 MWD, reduced C -reactive protein, and natriuretic peptides across all obesity classes in a dose-dependent manner, corroborate the role of WL in treating the pathobiology of obesity-related HFpEF [116]. Recently, treatment with tirzepatide (a long-acting agonist of glucose-dependent insulinotropic polypeptide and GLP-1R) led to a lower risk of a composite of death from CV causes or HF hospitalization and improved health status in patients with CM-HFpEF [117].

Finerenone, a non-steroidal, selective mineralocorticoid receptor antagonist (MRA), has recently emerged as an important treatment option for HFpEF, particularly in patients with CM comorbidities. Unlike steroidal MRAs (spironolactone, eplerenone), finerenone has greater receptor selectivity and a more balanced tissue distribution between the heart and kidneys, resulting in potent anti-inflammatory and anti-fibrotic effects with a more favorable safety profile (lower risk of hyperkalemia and renal impairment). In the FINerenone trial to investigate Efficacy and sAfety superioR to placebo in paTientS with Heart Failure (FINEARTS-HF) trial (n = 6001; LVEF ≥ 40%), it reduced the risk of worsening HF events and CV death by 16% [118]. Benefits were consistent across the full HFpEF spectrum and independent of diabetes status. It improved patient-reported QoL, with manageable hyperkalemia risk and no excess serious adverse events. Its benefit in CM-HFpEF likely relates to blocking aldosterone-driven inflammation and fibrosis, complementing SGLT2i and lifestyle therapy.

Interleukin-1 (IL-1) and IL-6 signaling represent promising anti-inflammatory targets in CM-HFpEF. Although early studies suggested improved exercise capacity with the IL-1 receptor antagonist anakinra, these findings were not confirmed in a larger phase 2 trial [119]. The ongoing HERMES trial is evaluating ziltivekimab (human monoclonal antibody targeting the IL-6 ligand) that reduces systemic inflammation in HFpEF [120].

## 15. How Do Current Heart Failure Guidelines Address CM-HFpEF?

Despite the rapidly growing prevalence of CM-HFpEF, its recognition as a distinct phenotype within major clinical guidelines remains limited but evolving. Both AHA/ACC/HFSA and ESC HF Guidelines acknowledge HFpEF heterogeneity and the role of metabolic comorbidities, though neither uses “CM-HFpEF” as a formal diagnostic category [36,121]. Among pharmacological therapies, SGLT-2i carry the strongest evidence base, with Class IIa recommendations in both guideline sets for reducing HF hospitalizations and CV death in HFpEF—an endorsement particularly relevant to the CM phenotype given the metabolic mechanisms discussed above [36,121]. Finerenone is not yet incorporated in current published guidelines, but the 2024 FINEARTS-HF data are expected to inform the next ESC and AHA updates; early expert consensus supports its consideration in eligible patients. GLP-1RAs are not yet included as an HF-specific therapy in HF guidelines, though they are recommended in T2DM and obesity guidelines where HF is a listed comorbidity.

Regarding lifestyle and non-pharmacological management, both guideline sets recommend WL for obese patients with HFpEF (Class IIa/IIb) and ET (Class IIa) to improve functional capacity and QoL [36,121]. Sodium restriction is listed as reasonable for symptomatic congestion, with acknowledgment of limited supporting trial data. Dietary modification following DASH or MedDiet patterns is encouraged as a comorbidity management strategy, though no HFpEF-specific dietary recommendations exist in either guideline. Both documents call for integrated management of all CM comorbidities—T2DM, HTN, dyslipidemia, and obesity—as central pillars of HFpEF care. A key gap remains that neither guideline provides a dedicated CM-HFpEF management pathway. Future iterations should incorporate phenotype-specific recommendations that prioritize metabolic therapies and structured lifestyle interventions as first-line disease-modifying strategies in this population.

## 16. What Are the Future Directions in CM-HFpEF?

Despite growing recognition of its clinical importance, CM-HFpEF remains inadequately defined beyond common metabolic comorbidities such as obesity and T2DM, limiting reproducibility across studies. Key gaps include the lack of integrated phenotypic frameworks that combine clinical features with imaging, circulating biomarkers, and functional measures to reliably distinguish CM-HFpEF from other HFpEF subtypes. Relatedly, optimal biomarker panels that reflect the dominant pathobiological axes—systemic inflammation, endothelial dysfunction, myocardial remodeling, and multiorgan crosstalk—remain undefined, and their utility for prognostication and therapeutic response stratification is uncertain. Therapeutically, while CM-HFpEF is conceptualized as a systemic inflammatory and metabolic disorder, the most effective strategies to target these interrelated pathways, particularly across the heart–adipose–skeletal muscle–renal axes, remain unclear.

ET is a cornerstone therapy in HFpEF; however, the optimal prescription (modality, intensity, and dose), durability of benefit, and effects on long-term clinical outcomes in CM-HFpEF remain insufficiently characterized. Moreover, adherence to ET represents a major barrier, with most studies limited to short-term interventions (3–6 months) and lacking a detailed understanding of behavioral, psychosocial, and social determinants of long-term maintenance, as well as methods to identify individuals at highest risk of non-adherence. In addition, strategies to effectively personalize and sustain lifestyle interventions—integrating exercise with dietary modification, pharmacologic therapies, and emerging digital health platforms—remain underdeveloped. Finally, multidisciplinary, integrated care models incorporating cardiology, endocrinology, nutrition, behavioral health, and physiotherapy may be required to optimize implementation and outcomes, but remain insufficiently tested in CM-HFpEF.

Together, these gaps highlight the need for integrated phenomapping and multi-omics approaches combined with advanced imaging and digital health-enabled longitudinal phenotyping to define CM-HFpEF endotypes and enable precision lifestyle and pharmacologic interventions.

### Take Home Message

- CM risk factors are fueling the rapid global expansion of CM- HFpEF phenotype.
- CM-HFpEF is a multisystem inflammatory-metabolic syndrome.
- CM-HFpEF shows greater symptom burden and biventricular adverse remodeling.
- Dietary modification and caloric restriction improve functional capacity.
- SGLT2i and GLP-1RAs are effective due to their metabolic protective effects.
- ET is a cornerstone intervention for HFpEF symptoms.

## 17. Conclusions

HFpEF has evolved from being viewed as an isolated disorder of DD to a complex systemic syndrome characterized by multisystem involvement and heterogeneous clinical phenotypes. Among these, the CM-HFpEF phenotype—defined by obesity, IR, HTN, and systemic inflammation—has emerged as the dominant and rapidly expanding form of HFpEF. The pathophysiology of this syndrome reflects the convergence of metabolic dysfunction, chronic systemic inflammation, coronary microvascular impairment, and maladaptive myocardial remodeling. Lifestyle interventions—including dietary modification, weight management strategies, and structured ET—directly address the upstream metabolic and inflammatory drivers of CM-HFpEF.

## Figures and Tables

**Table 1:** Oral supplementation of macro/micronutrients in patients with HFpEF.

Proteins	Protein supplementation (1.2 g/kg/day) alone failed to improve physical performance. However, when combined with light exercise (2 days of hydrotherapy and 1 day of gym sessions/week), there was significant improvement in some (6 min walk, 10 m walking speed, quadriceps strength) but not all physical function measurements [59].
Unsaturated fatty acids (UFA) (Pilot study)	Studies are limited. Only one small trial, with 84 days of consumption in 9 patients, showed improved exercise time and O_2_ pulse (limited by the small sample size and single-arm intervention) [60]. This pilot study counseled participants to consume at least 54 g of extra-virgin olive oil or canola oil, and/or 28 g of unsalted or lightly salted mixed nuts daily, and demonstrated the feasibility of dietary UFA supplementation.
Omega-3 Fatty Acids (RCT)	A greater n-3 index (the sum of eicosapentaenoic acid and docosahexaenoic acid content of red blood cell membrane) was associated with a favorable cardiac and metabolic profile and greater 6MWD and VO_2peak_ in patients with HFpEF. However, oral supplementation in patients with DD was not associated with improved cardiac function or body composition [61,62]. Linked to AF [63] Oral supplementation needs further safety evaluation.
Inorganic nitrate/Nitrite (RCT)	Inorganic nitrate supplementation, such as beetroot juice, can acutely improve VO_2peak._ However, chronic supplementation or nitrite therapies do not enhance submaximal endurance or long-term exercise capacity, indicating limited sustained clinical benefit [64].
Coenzyme Q10 (RCT) Pilot study	Short-term (1–4 months) dosing with 100 mg 3 times/day did not demonstrate improved DD, nor did 300 mg twice daily for 12 weeks; however, this higher dose did improve the Kansas City Cardiomyopathy Questionnaire clinical summary score, LVEF, and brain natriuretic peptide relative to placebo [65,66,67]. Overall, clinical evidence remains inconsistent, and a definitive benefit remains unproven [56,57].
L-carnitine (Pilot study)	It may aid weight loss and metabolic health, but evidence for improving diastolic function is lacking [68].
Vitamin D3 (RCT)	In 64 participants with HF, weekly 50,000 IU vitamin D3 for 6 months did not increase the primary outcome of VO_2peak_ compared with placebo, and adjustment for ejection fraction did not change the results [69].

Abbreviations: 6MWD = 6 min walk distance; AF = atrial fibrillation; DD = diastolic dysfunction; HF = heart failure; HFpEF = heart failure with preserved ejection fraction; IU = international units; LVEF = left ventricular ejection fraction; O_2_ = Oxygen; RCT = randomized controlled trial; UFA = unsaturated fatty acids; VO_2peak_ = peak oxygen consumption.

**Table 2:** Ongoing Dietary Intervention RCTs in Patients with HFpEF.

NCT#	Intervention	Primary Outcome/Study Completion Date
NCT05236413 (n = 36)	HIIT training versus DASH diet versus HIIT training and DASH diet, exercise supervised, all meals provided to participants, for 4 weeks	Change in VO_2peak_ Completed
NCT04235699 (n = 24)	Energy-restricted ketogenic diet versus energy-restricted low-fat diet, all meals provided to participants, for 4 weeks	Change in VO_2peak_ Completed
NCT06081543 (n = 90)	Ketogenic diet versus low-fat diet for 6 months—groceries provided for first 6 weeks, dietary counseling for duration of study	Change in VO_2peak_ August 2026
NCT06078683 (n = 30)	Ketone ester beverage or placebo beverage twice daily for 6 weeks, followed by a 4-week washout and 6 weeks of the alternate beverage	Change in VO_2peak_ August 2027
NCT05878912 (n = 120)	Energy-restricted diet—beginning with total meal replacement for 8 weeks with gradual food reintroduction vs. SOC control, lasting 3 to 6 months in total.	Change in left atrial volume index December 2026
NCT06044194 (n = 56)	L-arginine and liposomal vitamin C supplement vs. placebo for 3 months	Mitochondrial function in peripheral blood mononuclear cells Completed
NCT05887271 (n = 102)	Energy-restricted total meal replacement vs. attention control for 12 weeks	Change in 6MWD Completed

Abbreviations: 6MWD = 6 min walk distance; DASH = Dietary Approaches to Stop Hypertension; HFpEF = heart failure with preserved ejection fraction; HIIT = high-intensity interval training; NCT# = National Clinical Trial number; SOC = standard of care; VO_2peak_ = peak oxygen consumption.

**Table 3:** Clinical trials of Exercise therapy in HFpEF.

First Author/Trial (Ref. Number)	Intervention	HFpEF Patient Type	LVEF	Primary Endpoint	Trial Result
Gary et al. [81]	Exercise training (n = 32)	Aged 67 ± 11, all women, NYHA class II/III diastolic HF, ECHO–DD or diastolic HF, LVEF ≥ 45%	54 ± 7% (Mean ± SD)	6MWD	* Improved 6MWD * Quality-of-life and depression scores
PARIS/Kitzman et al. [82]	Exercise training (n = 53)	Aged 70 ± 6 years, 87% female, ambulatory HF patients with NYHA class II-III symptoms, LVEF ≥ 50%	61 ± 5% (Mean ± SD)	VO_2peak_	* Improved peak and submaximal exercise capacity This benefit was not associated with any measurable change in resting LV structure or function.
PARIS II/Haykowsky [83]	Exercise training (n = 40)	Aged 69 ± 6 years,82% female, ambulatory HF patients with NYHA class II-III symptoms, LVEF ≥ 50%	61 ± 5% (Mean ± SD)	VO_2peak_	* Improved VO_2peak_. Arterial-venous oxygen difference was the primary contributor to improved VO_2peak_
Kitzman et al. [84]	Exercise training (n = 63)	Aged 70 ± 7 years, 76% female, ambulatory HF patients with NYHA class II-III symptoms, LVEF ≥ 50%	58 ± 6% (Mean ± SD)	VO_2peak_, 6MWD	* Improved VO_2peak_ without altering endothelial function
SECRET-1/Kitzman et al. [48]	Caloric restriction and exercise training (n = 100)	Aged 67 ± 6 years, 80% female, ambulatory HF patients with NYHA class II-III symptoms LVEF ≥ 50%	60 ± 6% (Mean ± SD)	VO_2peak_, Quality of Life	* Increased VO_2peak_ and the effects may be additive * Quality of life by KCCQ was improved, and the benefit was greatest for caloric restriction
Ex-DHF trial [85]	Exercise training (n = 64)	Aged 65 ± 7 years,56% female, symptomatic, ambulatory NYHA II/III symptoms, echo-DD, LVEF ≥ 50%	68 ± 7% (Mean ± SD)	VO_2peak_	* Improved exercise capacity and quality of life scores by KCCQ This benefit was associated with atrial reverse remodeling and improved LV diastolic function
Smart et al. [86]	Exercise training (n = 25)	Aged 64 ± 8 years, 48% female, well compensated HF, LVEF > 45%	57 ± 10% (Mean ± SD)	VO_2peak_	Improved VO_2peak_ No significant changes in diastolic, systolic function, quality of life by KCCQ, and depression scores
Fu et al. [87]	Exercise training (n = 30)	Aged 61 ± 3 years, 33% female, NYHA class II/III HF, LVEF ≥ 50% with episodes of acute pulmonary edema.	58 ± 2% (Mean ± SD)	VO_2peak_	Improved VO_2peak_ Improved diastolic function with reduction of the E/e’ ratio Improved quality of life scores
Angadi SS et al. [88]	Exercise training (n = 9)	Aged 69 ± 6 years, 11% female, NYHA class II/III HF, ECHO-DD, LVEF ≥ 50%	65 ± 5% (Mean ± SD)	VO_2peak_	Improved VO_2peak_ Improved diastolic function
Alves et al. [89]	Exercise training (n = 31)	Aged 63 ± 11 years, 29% female, admission with clinical signs of HF. (LVEF > 55%)	56 ± 3% (Mean ± SD)	Exercise tolerance (METS), LVEF, and E/e′	* Improved exercise tolerance, cardiac systolic and diastolic function
Shaltout et al. [90]	Supervised aerobic exercise with vs. without dietary nitrate (n = 19)	69 ± 7 years, LVEF ≥ 50%, HFpEF (NYHA classes II-III)	Not reported	Submaximal aerobic endurance, measured as cycling time to exhaustion at 75% of measured maximal power output	* Both groups improved in submaximal aerobic endurance. There was no significant difference in submaximal aerobic endurance (the primary outcome) between the dietary nitrate and placebo groups.
REACH-HF/Lang et al. [91]	Comprehensive self-management rehabilitation program, including progressive exercise training program (n = 50)	72 ± 10 years, 64% female, HFpEF with LVEF ≥ 45%	Not reported	MLHFQ total score	* REACH-HF intervention improves exercise capacity and health-related quality of life. The program is feasible
Training-HF/Palau et al. [92]	IMT, FES, IMT + FES (n = 59)	Aged 74 ± 9 years, 58% female, clinically stable HFpEF, LVEF ≥ 50%, LVH/LAH or diastolic dysfunction, end-diastolic diameter < 60 mm	67 ± 10% (Mean ± SD)	VO_2peak_	* All interventional groups showed improved peak VO_2_ and quality of life. Effects were sustained at 24 weeks.
Azhar et al. [59]	ET + Protein, Protein only (n = 16)	Aged 70 ± 2 years, 50% female, HFpEF with LVEF ≥ 50%	58 ± 1% (Mean ± SD)	6MWD	* ET + Protein group showed improved 6MWD and blood pressure
Donelli da Silveira et al. [93]	HIIT, moderate continuous training (n = 19)	Aged 60 ± 9 years, 63% female, LVEF ≥ 50%, NYHA II–III	65 ± 5% (Mean ± SD)	VO_2peak_	* Improved VO_2peak_ in both groups, but more pronounced in HIIT group. * Improved diastolic function
Kinugasa et al. [94]	IMT (n = 20)	76 ± 10 years, 15% female, HFpEF with LVEF ≥ 45%	Not reported	Maximum inspiratory muscle pressure	* Improved maximum inspiratory muscle pressure and aerobic capacity
REHAB-HF/Kitzman et al. [95]	Tailored rehabilitation training (n = 185)	Aged 73 ± 9 years, 49% female, during or early after hospitalization for HF (HFpEF cohort had LVEF ≥ 45%)	Not reported	SPPB	* Improved physical function in multiple domains as measured by SPPB
Mueller et al. (OptimEx-CLIN) [79]	HIIT, MCT (n = 180)	HIIT: aged 70 ± 7 years, 71% female MCT: aged 70 ± 8 years, 60% female Control: aged 69 ± 10 years, 68% female LVEF ≥ 50%	HIIT: 62 ± 6% MCT: 62 ± 6% Control: 62 ± 5% (Mean ± SD)	VO_2peak_	Improvements in VO_2peak_ for both HIIT and MCT, without a significant difference between HIIT and MICT.
Alonso et al. (HEART camp) [96]	Multicomponent behavioral program including aerobic + resistance training with adherence support (n = 59)	Aged 64.6 ± 9.3 years, 44% female, HFpEF with LVEF ≥ 50%	55 ± 6% (Mean ± SD)	Adherence to exercise	* Improved adherence and functional capacity
SECRET-2/Brubaker et al. [49]	CR + AT, CR + AT + RT (n = 88)	Aged 70 ± 9 years, 85% female, chronic HFpEF, and BMI ≥ 28 kg/m^2^, LV EF ≥ 50%	61 ± 6% (Mean ± SD)	VO_2peak_	* CR + AT produces large improvements in VO_2peak_ and quality-of-life. Adding RT to CR + AT increased leg strength and muscle quality without attenuating skeletal muscle loss or further increasing VO_2peak_ or quality of life.
Liu et al. [97]	ET + pill, ET only (n = 60)	Aged 67 ± 7, 38% female, HFpEF with LVEF ≥ 50% and NYHA class II-III symptoms	Not reported	VO_2peak_	* ET improved VO_2peak_, anaerobic threshold, 6MWD, sleep, and quality of life
INABLE-training/Borlaug et al. [98]	ET + nitrites, ET + placebo (n = 92)	Aged 73 (66–76), 34% female, HFpEF with LVEF ≥ 50%	Mean: 60, Range: 55–64	VO_2peak_	* ET improved VO_2peak_ and quality of life No enhanced effect from nitrites
Obaya et al. [99]	Upper limb aerobic exercise, lower limb aerobic exercise (n = 48)	Aged 55 ± 7, 100% male, HFpEF with left ventricular end diastolic dimension > 5.5 cm and LVEF ≥ 50%	59 ± 4% (Mean ± SD)	VO_2peak_, LVEF	* Improved VO_2peak_ No change in LVEF
Sharif et al. [100]	RT (n = 24)	Aged 70 ± 7, 5% female, ambulatory HFpEF with LVEF ≥ 45%, NYHA class I-III	RT: 56 ± 5% Control: 54 ± 6 (Mean ± SD)	VO_2peak_	* Improved locomotor muscle composition, peak VO_2peak_ and muscle quality
Edelmann et al. [101]	Endurance ET (n = 322)	Aged 70 ± 7, 60% female, NYHA class II-III, clinically stable HFpEF with LVEF ≥ 50%	ET: 60 ± 5% Control: 62 ± 6%	Modified Packer score	* Improved VO_2peak_ and NYHA class Did not significantly improve the modified Packer score
Training-HR/Palau et al. [102]	AT, AT/LRT, AT/MCT to HIIT, ER (n = 80)	Aged 75 ± 7 years, 59.6% female, symptomatic (NYHA classes II-III/IV) patients with the ChI HFpEF phenotype, LVEF > 55%	65 ± 7% (Mean ± SD)	VO_2peak_	* All supervised training programs led to improvements in VO_2peak_ compared to ER

Abbreviations: AT = aerobic training; ChI = chronotropic incompetence; CR = caloric restriction; DD = diastolic dysfunction; E = mitral early diastolic velocity; ECHO = echocardiographically assessed; ER = non-supervised exercise recommendations; ET = exercise training; FES = functional electrical stimulation; HF = heart failure; HFpEF = heart failure with preserved ejection fraction; HIIT = high-intensity interval training; IMT = inspiratory muscle training; KCCQ = Kansas City Cardiomyopathy Questionnaire; LAH = left atrial hypertrophy; LRT = low-intensity strength training; LVH = left ventricular hypertrophy; LVEF = left ventricular ejection fraction; MCT = moderate continuous exercise; MLHFQ = Minnesota Living with Heart Failure Questionnaire; n = number of participants; NYHA = New York Heart Association; RT = resistance training; SD = standard deviation; SPPB = short physical performance battery; VO_2peak_ = peak oxygen consumption; 6MWD = 6 min walking distance. * Statistically significant and significant improvement.

## Data Availability

No new data created.
